# Facilitating pathway and network based analysis of RNA-Seq data with *pathlinkR*

**DOI:** 10.1371/journal.pcbi.1012422

**Published:** 2024-09-16

**Authors:** Travis M. Blimkie, Andy An, Robert E. W. Hancock

**Affiliations:** REW Hancock Laboratory, Center for Microbial Diseases and Immunity Research, Department of Microbiology and Immunology, University of British Columbia, Vancouver, British Columbia, Canada; University of Southern California, UNITED STATES OF AMERICA

## Abstract

R package *pathlinkR* is designed to aid transcriptomic analyses by streamlining and simplifying the process of analyzing and interpreting differentially expressed genes derived from human RNA-Seq data. It provides an integrated approach to performing pathway enrichment and network-based analyses, while also producing publication-quality figures to summarize these results, allowing users to more efficiently interpret their findings and extract biological meaning from large amounts of data. *pathlinkR* is available to install from the software repository Bioconductor at https://bioconductor.org/packages/pathlinkR/, with support available through the Bioconductor forums. The code, example, and supporting data is available on the GitHub repository at https://github.com/hancockinformatics/pathlinkR, under the GPL-3.0 license, where users may report problems or make suggestions using GitHub’s issue system.

## Introduction

RNA-Seq is one of the most prevalent types of omics methodologies, since it provides detailed insights into the biology underlying a variety of studies [1] through analysis of gene expression changes, and is applicable to any organism with a reference genome. Comparing one or more conditions of interest to a reference or control sample allows for the determination of genes that are undergoing changes in their expression specifically due to the condition or treatment. However, lists of differentially expressed genes (DEGs) by themselves are not sufficient for drawing meaningful conclusions about the mechanisms at work and they must be further analyzed. There are several paths one can choose with two of the most common approaches being pathway enrichment [2] and network analysis [3]; these can be combined to further extract biological meaning. While there are several tools and resources to perform these tasks [4,5], they tend to exist in separate packages, making their integration difficult, particularly for those not sufficiently trained in bioinformatics techniques.

Here we present *pathlinkR*, an R [6] package that provides a streamlined and unified interface to perform some of the most common analyses and visualizations of DEGs, with functions that can assist users to easily perform pathway enrichment and network analysis. It is designed to be straightforward to use and accepts input from the most commonly used primary tools in a typical analysis pipeline, *DESeq2* [7] and *edgeR* [8]. Both packages produce a table of DEGs, which *pathlinkR* can use without modification for input to its core functions, including visualizations, pathway enrichment, and construction of Protein-Protein Interaction (PPI) networks. These analyses can be performed on a number of databases, including Reactome, MSigDB, KEGG, and InnateDB (previously not available within an R package), which are each consistently formatted for simple incorporation into new analysis pipelines. For each of these analysis types supported by *pathlinkR*, users can generate publication-quality figures to summarize their findings.

## Design and Implementation

### Direct visualization of DEGs

Two functions in *pathlinkR* can be applied to a table of DEGs, enabling simple visualization and comparison of results; both can take standard data objects produced by DESeq2 [7] and edgeR [8] as input. The first is “*eruption*”, which creates a Volcano plot mapping fold change values on the x axis and statistical significance (negative log of the p value) on the y axis. Multiple refinements allow the resulting plot to be tweaked as desired, such as changing colours, adding a summary subtitle, and plotting fold changes as log-transformed or not. Selected genes can be highlighted by providing a list of gene names/IDs, or even the name of a pathway from which constituent genes are automatically highlighted. Pathways accessed by *pathlinkR* are from Reactome [9], a curated and peer-reviewed database of >2,600 pathways in *Homo sapiens*, as well as thousands of pathways from other model organisms, organized hierarchically based on cell-specific processes.

The second method for DEG visualization is “*plotFoldChange*,” which creates a heatmap with genes as rows and DE comparisons as the columns. This allows comparison of how the same set of genes behave in different comparisons of interest. The genes included in the rows can also be selected by providing a list of gene names/IDs, or the name of a Reactome pathway.

### Network analysis

A DEG table can be used as input to “*ppiBuildNetwork*” to create a PPI network, using curated molecular human interaction data from InnateDB [10] (previously not available within the R framework), an IMEX consortium database, and leveraging functions from the igraph [11] and tidygraph [12] packages. Options for network order including zero, first, or minimum are available to suit the number of genes used as a seed for the network. A zero order network contains only genes or nodes that are input by the user, referred to as seed nodes—no additional genes are added via interactions. First order networks include genes which interact with the seed nodes, called interactors, growing and expanding the network using the available data. A minimum order network starts as first order, but any interactors with only a single connection to a seed node are removed, trimming down the overall network. Once built, this network can be visualized directly within R via “*ppiPlotNetwork*,” which can easily map a variable of interest (e.g. fold change directionality, statistical significance, or node degree) to node colour, and highlight and label key “hub” nodes which are integral in binding the network together. PPI networks can be easily tested for enriched pathways using “*ppiEnrichNetwork*” (it uses the same methodology as the main pathway enrichment function; see the next section for details) and a sub-network consisting of nodes present in a pathway of interest can be plotted by using “*ppiExtractSubnetwork*,” followed by the same “*ppiPlotNetwork*” function to create an updated graph highlighting nodes from the pathway. These methods are specifically designed to work with the results from human RNA-Seq experiments, providing previously unavailable network functionality in the context of analyzing DEGs.

### Pathway enrichment

The “*pathwayEnrichment*” function can be used on a list of one or more DEG tables, testing each for enriched pathways, and returning a single table to facilitate simple comparison of results, making pathlinkR inherently scalable to complex experiments. Three methodologies for enrichment are included: The first is simple over-representation analysis, utilized by the popular *ReactomePA* [4] package, which treats genes in a pathway individually when calculating the statistical significance of enrichment. The second is gene-pair over-representation analysis, utilized by the *Sigora* package [13], which treats genes in a pathway as unique pairs to provide less redundant pathway enrichment results. Both options are available when analyzing the Reactome [9] or KEGG [14] human pathway databases, while a second option uses simple over-representation analysis with the Molecular Signatures Database (MSigDB) Hallmark Collection [15] for humans, which are gene sets that represent “specific, well-defined biological states or processes with coherent expression”. Finally, users may also perform gene set enrichment analysis (GSEA) built on the *fgsea* [16] package, on either Reactome pathways or MSigDB Hallmark terms. These methods have been provided to enable users to have more freedom in determining which methodology and database suits their analysis. Unlike most pathway enrichment tools, for “pathwayEnrichment” the input DEGs from a single comparison can be treated as a complete set, or split based on the directionality of each gene’s fold change, resulting in pathways assigned as “upregulated” (i.e. enriched by upregulated genes, meaning the pathway overall can be thought of as being activated) or “downregulated.” The output format is shared for all available methods and databases, making it simple to incorporate any of these tests into a larger pipeline.

The table of results from “*pathwayEnrichment*” can be visualized with “*pathwayPlots*” to create a compelling figure which summarizes and compares the enrichment results across multiple DEG comparisons, scaling to experiments with multiple conditions of interest. Each comparison (e.g. Treatment A vs. Control and Treatment B vs. Control) is represented as a column on the x axis, while significant pathways are placed alphabetically as the rows on the y axis. A triangle indicates a given pathway was significantly enriched in one comparison, while its direction (pointing up or down) denotes if the pathway was derived from up- or down-regulated genes; or if there is no separation by directionality, circles are used instead. When using any of the methods or databases available, the pathways on the y axis can be grouped according to higher level categories, such as “Immune System” or “Metabolism,” to increase interpretability. A number of options can be used to tweak and customize the final output, such as only showing pathways from certain categories, or adding the number of DEGs below each comparisons’ name on the x axis.

### Pathway-based networks

The pathway enrichment results from a single DE comparison can be visualized as a network using the “*pathnet*” family of functions. Each pathway becomes a node in a network, with interactions or edges defined by the number of shared genes between two pathways. Since many genes can have multiple functions and play a role in different pathways, this type of analysis provides an alternative method of visualization that groups seemingly unrelated pathways based on their shared constituent genes, identifying similarities that may otherwise be overlooked. To determine if two pathways are connected, a number of standard distance measures are supported (e.g. Jaccard, Euclidean, and Manhattan), and a threshold is set to define a pair of pathways as being interconnected. By default, the Jaccard Index is used to measure the distance of pathway pairs, and a threshold of 0.8 is used to define a pathway pair as connected. Communities or subgraphs of nodes are not strictly defined, but rather arise organically based on the methods and options set by the user. Two options are provided for visualizing these pathway-based networks within R. The first produces a static plot of the network using the ggraph [17] library; the second uses visNetwork [18] and is interactive, allowing nodes to be selected, dragged, and grouped together, or categories of nodes (e.g. “Immune System”) can be highlighted—all within the R interface. This dynamic plot can be saved as a standard image file for inclusion in a summary presentation or publication.

## Results

**Fig 1:**
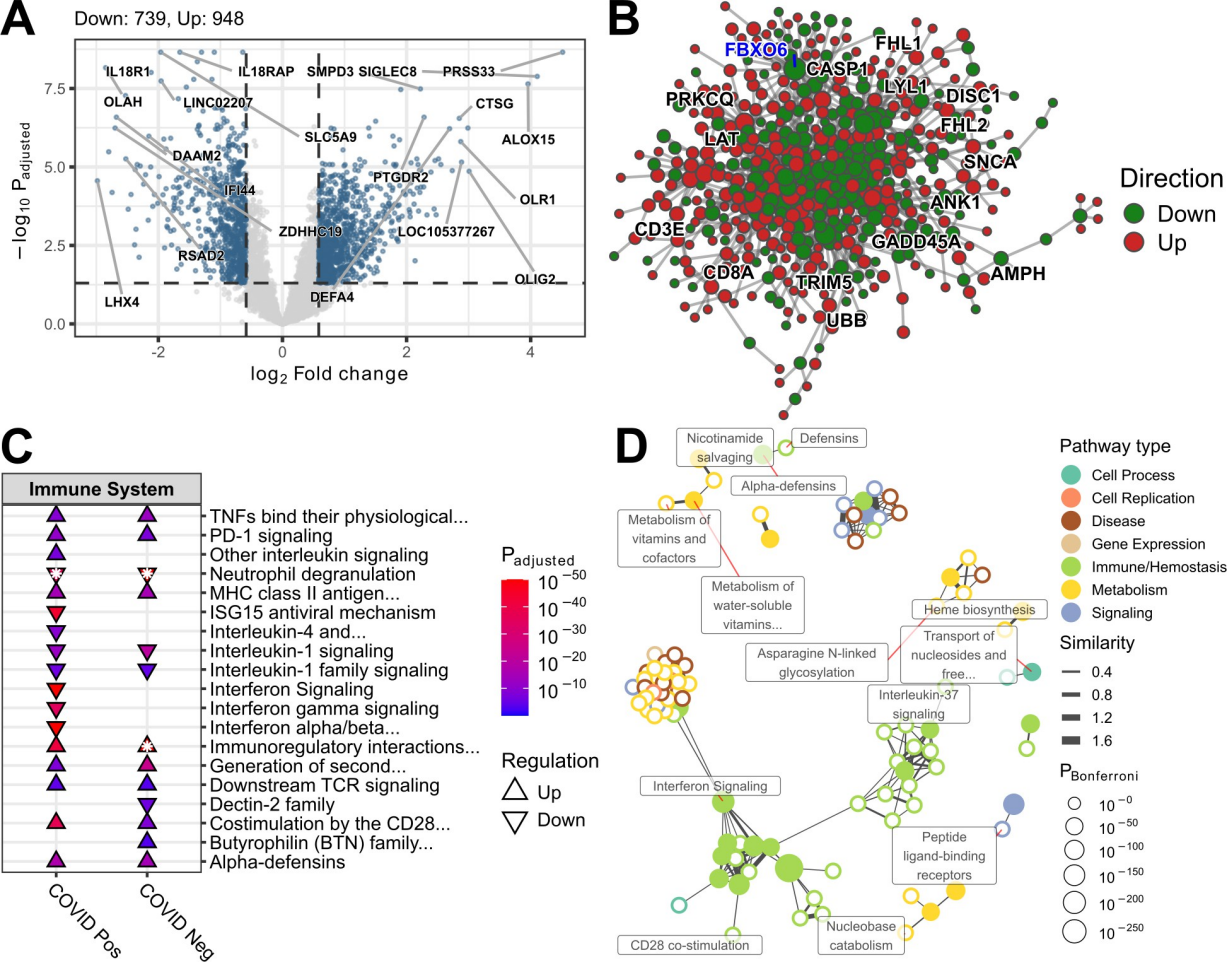
A selection of figures summarizing results generated by *pathlinkR*. **A:** Volcano plot created by “*eruption*” showing the fold changes and significance of DEGs, automatically labelling the top genes. **B:** A zero order PPI network created with *pathlinkR* using PPI data from InnateDB, with the node colour indicating fold change direction, and key hub nodes highlighted with blue **or black** labels.. **C:** Plotting the results of “*pathwayEnrichment*” for immune pathways, with triangles to indicate the presence and directionality of pathways for each condition on the x axis, and pathways sorted alphabetically on the y-axis. An asterisk indicates a pathway was significant in both directions; only one triangle is depicted, corresponding to the direction with the lower p value. **D:** A network constructed from pathway enrichment results, with nodes representing single pathways, and edges drawn for pathways with similarity above a specified threshold.

The example data set provided by pathlinkR is derived from An et al. [19], which details the study of COVID-19 positive and negative sepsis patients at admission compared to one week later in the ICU. The analysis begins by creating a volcano plot of the DEGs from the COVID-19 positive group (one week vs. admission, **Fig 1A**). Here it can be observed that a substantial number of genes are differentially expressed, primarily upregulated, as indicated in the automatically included title above the plot. By default a selection of top genes ranked by absolute fold change and adjusted p-value are labeled using their HGNC symbols.

To further explore these genes and their potential role in disease, a PPI network can be created using InnateDB interaction data supplied by *pathlinkR*, and providing the DEGs from COVID-19 positive sepsis patients as seed nodes for a zero order network (**Fig 1B**). The resultant network is densely connected and composed of a mixture of up and down regulated genes, with key hubs highlighted in blue, **or black** including immune genes CDH1 and HECW2.

To compare the transcriptional changes in COVID-19 positive and negative sepsis patients over time, pathway enrichment can be performed on these two lists of DEGs using “pathwayEnrichment” and the *Sigora* method of gene-pair analysis on the Reactome pathway database. The default when performing pathway enrichment is to treat up and downregulated genes independently for each comparison, and recombine the results at the end. Then the significantly enriched pathways are visualized using “plotPathways” to produce the plot shown in **Fig 1C**. Attention can be focused by e.g. specifying that only immune-related pathways should be included, making it easier to identify differences in the two results, such as the lack of enriched interferon pathways in the COVID-19 negative sepsis patients compared to their upregulation in the COVID-19 positive sepsis patients.

Lastly, the enriched pathways derived from the COVID-19 positive patients can be used to create a pathway network or “pathnet” using the provided functionality to generate a pathway distance matrix, create the pathway network “foundation” (i.e. interacting pathways), then creating the pathway network itself. Two means of visualizing these pathway networks are provided, one static and one interactive. **Fig 1D** shows the static pathway network, where each node is a pathway, and edges are drawn if the distance between two pathways (defined by their overlapping genes) is below a defined threshold. In this example, Jaccard distance was used to define pathway interactions, and a distance threshold of 0.8 was set to determine pairs of interacting pathways. Filled nodes represent pathways that were significantly enriched, while empty nodes are those interactors that did not meet statistical significance. Each node is coloured according to its category, the same as those used in **Fig 1C** to plot only immune pathways. This type of visualization allows the identification of groups of similar pathways that may be overlooked, such as MAP kinase cascade pathways and disease and immune pathways, including interleukin signaling.

## Supporting information

S1 SourceCode Source package of pathlinkR, version 1.1.13, representing the “devel” Bioconductor branch 3.20 and including changes requested by reviewers during the revision process. (TAR.GZ)
