# Supplementary material for: Facilitating pathway and network based analysis of RNA-Seq data with pathlinkR
Source: PLoS Comput Biol. 2024 Sep 16;20(9):e1012422. doi: 10.1371/journal.pcbi.1012422 (PMC11404784; doi:10.1371/journal.pcbi.1012422)
Supplement: S1 SourceCode — (TAR.GZ) [file pcbi.1012422.s001.tar.gz › pathlinkR/inst/doc/pathlinkR.html]

Analyze and visualize RNA-Seq data with pathlinkR


# Analyze and visualize RNA-Seq data with pathlinkR

Andy An and Travis Blimkie

#### 21 July 2024

# Contents

- 1 Introduction
- 2 Installation
- 3 Visualizing RNA-Seq data with volcano plots
- 4 Visualizing fold changes across comparisons
- 5 Building and visualizing PPI networks
  - 5.1 Enriching networks and extracting subnetworks
- 6 Performing pathway enrichment
- 7 Plotting pathway enrichment results
- 8 Generating networks from enriched pathways
- 9 Supplemental materials
  - 9.1 Gene-pair signatures
  - 9.2 Why are there different p-value cut-offs for sigora vs. ReactomePA/Hallmark?
- 10 Citations
- 11 Session information


This vignette contains some minimal examples for the main **pathlinkR**
functions; for more complete documentation, please see our
Github pages.

# 1 Introduction

Often times, gene expression studies such as microarrays and RNA-Seq result in
hundreds to thousands of differentially expressed genes (DEGs). It becomes very
difficult to understand the biological significance of such massive data sets,
especially when there are multiple conditions and comparisons being analyzed.
This package facilitates visualization and downstream analyses of differential
gene expression results, using pathway enrichment and protein-protein
interaction networks, to aid researchers in uncovering underlying biology and
pathophysiology from their gene expression studies.

We have included an example data set of gene expression results in this package
as the object `exampleDESeqResults`. This is a list of 2 data frames, generated
using the `results()` functions from the package
*DESeq2* (Love et al. 2014). The data is from an RNA-Seq
study investigating COVID-19 and non-COVID-19 sepsis patients at admission (T1)
compared to approximately1 week later (T2) in the ICU, indexed over time (i.e.,
T2 vs T1) (An et al. 2023).

# 2 Installation

To install and load the package:

```
# We'll also be using some functions from dplyr
# BiocManager::install("pathlinkR", version="devel")
library(dplyr)
library(pathlinkR)
```

# 3 Visualizing RNA-Seq data with volcano plots

One of the first visualizations commonly performed with gene expression studies
is to identify the number of DEGs. These are typically defined using specific
cutoffs for both fold change and statistical significance. Thresholds of
adjusted p-value <0.05 and absolute fold change >1.5 are used as the default,
though any value can be specified. **pathlinkR** includes the function
`eruption()` to create a volcano plot.

```
## A quick look at the DESeq2 results table
data("exampleDESeqResults")
knitr::kable(head(exampleDESeqResults[[1]]))
```

|  | baseMean | log2FoldChange | lfcSE | stat | pvalue | padj |
| --- | --- | --- | --- | --- | --- | --- |
| ENSG00000000938 | 16292.64814 | -0.5624954 | 0.1458274 | -3.857268 | 0.0001147 | 0.0013531 |
| ENSG00000002586 | 1719.51750 | 0.4501181 | 0.1520122 | 2.961066 | 0.0030658 | 0.0153820 |
| ENSG00000002919 | 870.64168 | -0.2445729 | 0.1249293 | -1.957690 | 0.0502664 | 0.1236844 |
| ENSG00000002933 | 266.65476 | 0.8838310 | 0.2093628 | 4.221528 | 0.0000243 | 0.0004313 |
| ENSG00000003249 | 11.43282 | 1.3287128 | 0.2881385 | 4.611369 | 0.0000040 | 0.0001200 |
| ENSG00000003509 | 207.88545 | -0.1825614 | 0.1763556 | -1.035189 | 0.3005807 | 0.4453252 |

```
## Generate a volcano plot from the first data frame, with default thresholds
eruption(
    rnaseqResult=exampleDESeqResults[[1]],
    title=names(exampleDESeqResults[1])
)
```

There are multiple options available for customizing this volcano plot,
including:

- Adjust the cutoffs
- Switch the colours of significant and non-significant genes
- Change the number of labelled genes
- Adjust the x- and y-axis limits
- Plot either log2 or non-log2 fold change (for a better sense of magnitude)
- Highlight specific gene sets of interest

# 4 Visualizing fold changes across comparisons

In addition to creating volcano plots, we can also visualize our DEGs using
heatmaps of genes involved in a specific pathways (e.g. one identified as
significant by `pathwayEnrichment()`). The function `plotFoldChange()`
accomplishes this by taking in an input list of `DESeq2::results()` data frames,
just like `pathwayEnrichment()`, and creating a heatmap of fold changes for the
constituent genes.

```
plotFoldChange(
    inputList=exampleDESeqResults,
    pathName="Interferon alpha/beta signaling"
)
```

A number of options are provided for customization, including:

- Shorten the names of each comparison
- Provide a custom list of genes to visualize, and add an informative title
- Use log2 fold changes in the heatmap cells
- Swap the rows and columns from the default, and add a split between the rows
  (comparisons)

# 5 Building and visualizing PPI networks

**pathlinkR** includes tools for constructing and visualizing Protein-Protein
Interaction (PPI) networks. Here we leverage PPI data gathered from
InnateDB to generate a list of interactions among
DE genes identified in gene expression analyses. These interactions can then be
used to build PPI networks within R, with multiple options for controlling the
type of network, such as support for first, minimum, or zero order networks. The
two main functions used to accomplish this are `ppiBuildNetwork()` and
`ppiPlotNetwork()`.

Let’s continue looking at the DEGs from the COVID positive patients over time,
using the significant DEGs to build a PPI network. Since the data frame we’re
inputting includes all measured genes (not just the significant ones), we’ll use
the `filterInput=TRUE` option to ensure the network is made only with those
genes which pass the standard thresholds (defined above). Since we’re
visualizing a network of DEGs, let’s colour the nodes to indicate the direction
of their dysregulation (i.e. up- or down-regulated) by specifying
`fillType="foldChange"`.

```
exNetwork <- ppiBuildNetwork(
    rnaseqResult=exampleDESeqResults[[1]],
    filterInput=TRUE,
    order="zero"
)

ppiPlotNetwork(
    network=exNetwork,
    title=names(exampleDESeqResults)[1],
    fillColumn=LogFoldChange,
    fillType="foldChange",
    label=TRUE,
    labelColumn=hgncSymbol,
    legend=TRUE
)
```

The nodes with blue labels (e.g. STAT1, FBXO6, CDH1, etc.) are hubs within the
network; i.e. those genes which have a high betweenness score. The statistic
used to determine hub nodes can be set in `ppiBuildNetwork()` with the
“hubMeasure” option.

## 5.1 Enriching networks and extracting subnetworks

**pathlinkR** includes two functions for further analyzing PPI networks. First,
`ppiEnrichNetwork()` will use the node table from a network to test for enriched
Reactome pathways or Hallmark gene sets (see the next section for more detail on
the pathway enrichment methods):

```
exNetworkPathways <- ppiEnrichNetwork(
    network=exNetwork,
    analysis="hallmark",
    filterResults="default",
    geneUniverse = rownames(exampleDESeqResults[[1]])
)
```

Second, the function `ppiExtractSubnetwork()` can extract a minimally-connected
subnetwork from a starting network, using the genes from an enriched pathway as
the “starting” nodes for extraction. For example, below we use the results from
the Hallmark enrichment above to pull out a subnetwork of genes from the
“Interferon Gamma Response” term, then plot this reduced network while
highlighting the genes from the pathway:

```
exSubnetwork <- ppiExtractSubnetwork(
    network=exNetwork,
    pathwayEnrichmentResult=exNetworkPathways,
    pathwayToExtract="INTERFERON GAMMA RESPONSE"
)

ppiPlotNetwork(
    network=exSubnetwork,
    fillType="oneSided",
    fillColumn=degree,
    label=TRUE,
    labelColumn=hgncSymbol,
    legendTitle="Degree"
)
```

Alternatively you can use the “genesToExtract” argument in
`ppiExtractSubnetwork()` to supply your own set of genes (as Ensembl IDs) to
extract as a subnetwork.

# 6 Performing pathway enrichment

The essence of pathway enrichment is the concept of over-representation: that
is, are there more genes belonging to a specific pathway present in our DEG list
than we would expect to find by chance? To calculate this, the simplest method
is to compare the ratio of DEGs in some pathway to all DEGs, and all genes in
tat pathway to all genes in all pathways in a database. **pathlinkR** mainly
uses the Reactome database (Fabregat et al. 2017) for
this purpose.

One issue that can occur with over-representation analysis is the assumption
that each gene in each pathway has “equal” value in belonging to that pathway.
In reality, a single protein can have multiple (and sometimes very different)
functions, and belong to multiple pathways, like protein kinases. There are also
pathways that have substantial overlap with cellular machinery, like the TLR
pathways. This can lead to enrichment of multiple similar pathways or even
unrelated “false-positives” that make parsing through the results very
difficult.

One solution is to use unique gene pairs, as described by the creators of the
package *sigora* (Foroushani et al.
2013). This methodology decreases the number of similar and unrelated pathways
from promiscuous genes, focusing more on the pathways that are likely related to
the underlying biology. This approach is the default used in the **pathlinkR**
function `pathwayEnrichment()`.

The `pathwayEnrichment()` function takes as input a list of data frames (each
from `DESeq2::results()`), and by default will split the genes into up- and
down-regulated before performing pathway enrichment one each set. The name of
the data frames in the list should indicate the comparison that was made in the
DESeq2 results, as it will be used to identify the results. For
`analysis="sigora"` we also need to provide a Gene Pair Signature Repository
(`gpsRepo`) which contains the pathways and gene pairs to be tested. Leaving
this argument to “default” will use the `reaH` GPS repository from
**sigora**, containing human Reactome pathways. Alternatively one can supply
their own GPS repository; see `??sigora::makeGPS()` for details on how to
make one.

```
enrichedResultsSigora <- pathwayEnrichment(
    inputList=exampleDESeqResults,
    analysis="sigora",
    filterInput=TRUE,
    gpsRepo="default"
)

head(enrichedResultsSigora)
```

```
# A tibble: 6 × 12
  comparison       direction pathwayId pathwayName   pValue pValueAdjusted genes
  <chr>            <chr>     <chr>     <fct>          <dbl>          <dbl> <chr>
1 COVID Pos Over … Up        R-HSA-38… Chemokine … 7.04e-46       7.05e-43 CCR3…
2 COVID Pos Over … Up        R-HSA-19… Immunoregu… 5.46e-45       5.46e-42 CD1A…
3 COVID Pos Over … Up        R-HSA-38… Costimulat… 2.56e-40       2.56e-37 CD28…
4 COVID Pos Over … Up        R-HSA-67… Neutrophil… 7.88e-31       7.89e-28 ABCA…
5 COVID Pos Over … Up        R-HSA-20… Cell surfa… 3.55e-21       3.55e-18 ATP1…
6 COVID Pos Over … Up        R-HSA-14… Alpha-defe… 1.02e-20       1.02e-17 CD4;…
# ℹ 5 more variables: numCandidateGenes <dbl>, numBgGenes <int>,
#   geneRatio <dbl>, totalGenes <int>, topLevelPathway <chr>
```

For those who still prefer traditional over-representation analysis, we include
the option of doing so by setting `analysis="reactome"`, which uses
*ReactomePA* (Yu et al. 2016). When using this method,
we recommend providing a gene universe to serve as a background for the
enrichment test; here we’ll use all the genes which were tested for significance
by DESeq2 (i.e. all genes from the count matrix), converting them to Entrez gene
IDs before running the test. See the full vignette at our
Github pages for details.

In addition to the Reactome database used when setting `analysis` to “sigora” or
“reactome”, we also provide over-representation analysis using the Hallmark
gene sets from
the Molecular Signatures Database (MSigDb). These are 50 gene sets that
represent “specific, well-defined biological states or processes with coherent
expression” (Liberzon et al. 2015). This database provides a more high-level
summary of key biological processes compared to the more granular Reactome
pathways.

```
enrichedResultsHm <- pathwayEnrichment(
    inputList=exampleDESeqResults,
    analysis="hallmark",
    filterInput=TRUE,
    split=TRUE
)

head(enrichedResultsHm)
```

```
# A tibble: 6 × 12
  comparison       direction pathwayId pathwayName   pValue pValueAdjusted genes
  <chr>            <chr>     <chr>     <chr>          <dbl>          <dbl> <chr>
1 COVID Pos Over … Up        HEME MET… HEME METAB… 2.69e-32       1.34e-30 SLC4…
2 COVID Pos Over … Up        IL2 STAT… IL2 STAT5 … 5.89e- 4       1.47e- 2 PLPP…
3 COVID Pos Over … Down      INTERFER… INTERFERON… 3.14e-30       1.48e-28 EIF2…
4 COVID Pos Over … Down      INTERFER… INTERFERON… 2.37e-28       5.58e-27 EIF2…
5 COVID Pos Over … Down      INFLAMMA… INFLAMMATO… 6.83e- 9       1.07e- 7 KIF1…
6 COVID Pos Over … Down      TNFA SIG… TNFA SIGNA… 1.75e- 7       2.06e- 6 MXD1…
# ℹ 5 more variables: numCandidateGenes <dbl>, numBgGenes <dbl>,
#   geneRatio <dbl>, totalGenes <int>, topLevelPathway <chr>
```

Finally, users may also use data from KEGG when
performing enrichment analyses. This database is supported with traditional
over-representation analysis by setting `analysis="kegg"`, or with a gene
pair-based approach by specifying `analysis="sigora"` and `gpsRepo="kegH"`.

# 7 Plotting pathway enrichment results

Now that we have (a lot of) pathway enrichment results from multiple
comparisons, its time to visualize them. The function `plotPathways()` does this
by grouping Reactome pathways (or Hallmark gene sets) under parent groups, and
indicates if each pathway is up- or down-regulated in each comparison, making it
easy to identify which pathways are shared or unique to different DEG lists.
Because there are often many pathways, you can split the plot into multiple
columns (up to 3), and truncate the pathway names to make the results fit more
easily.

Sometimes a pathway may be enriched in both up- and down-regulated genes from
the same DEG list (these usually occur with larger pathways). Such occurrences
are indicated by a white asterisk where the more significant (lower adjusted
p-value) direction is displayed. You can also change the angle/labels of the
comparisons, or add the number of DEGs in each comparison below the labels.
Lastly, you can specify which pathways or top pathway groups to include for
visualization.

```
pathwayPlots(
    pathwayEnrichmentResults=enrichedResultsSigora, 
    columns=2
)
```

A variety of tweaks can be applied to these plot as well:

- Only show immune-related pathways based on the top pathway grouping
- Change the colour scaling used for the adjusted p-values
- Condense the comparison names to better fit onto the plot, and make them
  display horizontally
- Add the number of DEGs used for enrichment below the comparison name
- Increase the truncation cutoff value of pathway names for more words

From these results, you can see that while many of the immune system pathways
change in the same direction over time in COVID-19 and non-COVID-19 sepsis
patients, a few unique ones stand out, mostly related to interferon signaling
(“Interferon Signaling”, “Interferon gamma signaling”, “Interferon alpha/beta
signaling”, “ISG15 antiviral mechanism”). This likely reflects an elevated early
antiviral response in COVID-19 patients that decreased over time, compared to no
change in non-COVID-19 sepsis patients.

# 8 Generating networks from enriched pathways

**pathlinkR** includes functions for turning the pathway enrichment results
from either Reactome-based method (“sigora” or “reactomepa”) into networks,
using the overlap of the genes assigned to each pathway to determine their
similarity to one other. In these networks, each pathway is a node, with
connections or edges between them determined via a distance measure. A threshold
can be set, where two pathways with a minimum similarity measure are considered
connected, and would have an edge drawn between their nodes.

We provide a pre-computed distance matrix of Reactome pathways, generated using
Jaccard distance, but there is support for multiple distance measures to be
used. Once this “foundation” of pathway interactions is created, a pathway
network can be built using the `createPathnet()` function:

```
data("sigoraDatabase")

pathwayDistancesJaccard <- getPathwayDistances(pathwayData = sigoraDatabase)

startingPathways <- pathnetFoundation(
    mat=pathwayDistancesJaccard,
    maxDistance=0.8
)

# Get the enriched pathways from the "COVID Pos Over Time" comparison
exPathwayNetworkInput <- enrichedResultsSigora %>% 
    filter(comparison == "COVID Pos Over Time")

myPathwayNetwork <- pathnetCreate(
    pathwayEnrichmentResult=exPathwayNetworkInput,
    foundation=startingPathways
)
```

There are two options for visualization, the first being a static network:

```
pathnetGGraph(
    myPathwayNetwork,
    labelProp=0.1,
    nodeLabelSize=3,
    nodeLabelOverlaps=8,
    segColour="red",
    themeBaseSize = 12
)
```

Nodes (pathways) which are filled in are enriched pathways (i.e. those output by
`pathwayEnrichment()`). Size of nodes is correlated with statistical
significance, while edge thickness relates to the similarity of two connected
pathways.

Though this type of visualization is useful, we can also display this network
using an alternate method that creates an interactive display, with the function
`pathnetVisNetwork()`; see our
Github pages for these
details.

# 9 Supplemental materials

## 9.1 Gene-pair signatures

**sigora** uses a Gene-Pair Signature (GPS) Repository that stores information
on which gene pairs are unique for which pathways. We recommend using the one
provided by sigora, which can be loaded via `data("reaH", package = "sigora")`
(Reactome Human). You can also generate your own GPS repo using sigora’s own
function and a custom set of pathways (e.g. from another pathway database like
GO or KEGG). Please consult the sigora documentation on how to generate your
custom GPS repository.

## 9.2 Why are there different p-value cut-offs for sigora vs. ReactomePA/Hallmark?

Because there are now multiple gene pairs vs. single genes, the gene-pair
“universe” is greatly increased and it is more likely for a result to be
significant. Therefore, the cutoff threshold for significance is more stringent
(adjusted p-value < 0.001) and a more conservative adjustment method
(Bonferroni) is used. For regular over-representation analysis, a less
conservative adjustment method (Benjamini-Hochberg) is used with adjusted
p-value < 0.05. These are automatically set with `filterResults="default"`. You
can adjust these cut-offs by setting `filterResults` to different values between
0 and 1, or 1 if you want all the pathways (this may be useful for comparing
which enriched genes appear in which comparisons, even if the enrichment is not
significant).

# 10 Citations

An AY, Baghela AS, Falsafi R, Lee AH, Trahtemberg U, Baker AJ, dos Santos CC,
Hancock REW. Severe COVID-19 and non-COVID-19 severe sepsis converge
transcriptionally after a week in the intensive care unit, indicating common
disease mechanisms. Front Immunol. 2023;6(14):1167917.

Fabregat A, Sidiropoulos K, Viteri G, Forner O, Marin-Garcia P, Arnau V,
D’Eustachio P, Stein L, Hermjakob H. Reactome pathway analysis: a
high-performance in-memory approach. BMC Bioinform. 2017;18:142.

Foroushani ABK, Brinkman FSL, Lynn DJ. Pathway-GPS and sigora: identifying
relevant pathways based on the over-representation of their gene-pair
signatures. PeerJ. 2013;1:e229.

Liberzon A, Birger C, Thorvaldsdóttir H, Ghandi M, Mesirov JP, Tamayo P. The
Molecular Signatures Database (MSigDB) hallmark gene set collection. Cell Syst.
2015;1(6):417–25.

Love MI, Huber W, Anders S. Moderated estimation of fold change and dispersion
for RNA-seq data with DESeq2. Genome Biol. 2014;15(12):550.

Yu G, He QY. ReactomePA: an R/Bioconductor package for reactome pathway analysis
and visualization. Mol Biosyst. 2016;12(2):477-9.

# 11 Session information

```
R version 4.4.1 (2024-06-14)
Platform: x86_64-pc-linux-gnu
Running under: Ubuntu 22.04.4 LTS

Matrix products: default
BLAS:   /home/biocbuild/bbs-3.20-bioc/R/lib/libRblas.so 
LAPACK: /usr/lib/x86_64-linux-gnu/lapack/liblapack.so.3.10.0

locale:
 [1] LC_CTYPE=en_US.UTF-8       LC_NUMERIC=C              
 [3] LC_TIME=en_GB              LC_COLLATE=C              
 [5] LC_MONETARY=en_US.UTF-8    LC_MESSAGES=en_US.UTF-8   
 [7] LC_PAPER=en_US.UTF-8       LC_NAME=C                 
 [9] LC_ADDRESS=C               LC_TELEPHONE=C            
[11] LC_MEASUREMENT=en_US.UTF-8 LC_IDENTIFICATION=C       

time zone: America/New_York
tzcode source: system (glibc)

attached base packages:
[1] stats4    stats     graphics  grDevices utils     datasets  methods  
[8] base     

other attached packages:
 [1] DESeq2_1.45.0               SummarizedExperiment_1.35.1
 [3] Biobase_2.65.0              MatrixGenerics_1.17.0      
 [5] matrixStats_1.3.0           GenomicRanges_1.57.1       
 [7] GenomeInfoDb_1.41.1         IRanges_2.39.2             
 [9] S4Vectors_0.43.2            BiocGenerics_0.51.0        
[11] pathlinkR_1.1.13            dplyr_1.1.4                
[13] BiocStyle_2.33.1           

loaded via a namespace (and not attached):
  [1] RColorBrewer_1.1-3      jsonlite_1.8.8          shape_1.4.6.1          
  [4] magrittr_2.0.3          magick_2.8.4            farver_2.1.2           
  [7] rmarkdown_2.27          GlobalOptions_0.1.2     fs_1.6.4               
 [10] zlibbioc_1.51.1         vctrs_0.6.5             memoise_2.0.1          
 [13] Cairo_1.6-2             ggtree_3.13.0           rstatix_0.7.2          
 [16] tinytex_0.52            htmltools_0.5.8.1       S4Arrays_1.5.5         
 [19] broom_1.0.6             gridGraphics_0.5-1      SparseArray_1.5.25     
 [22] sass_0.4.9              bslib_0.7.0             htmlwidgets_1.6.4      
 [25] plyr_1.8.9              cachem_1.1.0            igraph_2.0.3           
 [28] lifecycle_1.0.4         iterators_1.0.14        pkgconfig_2.0.3        
 [31] gson_0.1.0              Matrix_1.7-0            R6_2.5.1               
 [34] fastmap_1.2.0           GenomeInfoDbData_1.2.12 clue_0.3-65            
 [37] aplot_0.2.3             enrichplot_1.25.0       digest_0.6.36          
 [40] colorspace_2.1-0        patchwork_1.2.0         AnnotationDbi_1.67.0   
 [43] RSQLite_2.3.7           ggpubr_0.6.0            vegan_2.6-6.1          
 [46] labeling_0.4.3          fansi_1.0.6             httr_1.4.7             
 [49] polyclip_1.10-6         abind_1.4-5             mgcv_1.9-1             
 [52] compiler_4.4.1          bit64_4.0.5             withr_3.0.0            
 [55] doParallel_1.0.17       backports_1.5.0         BiocParallel_1.39.0    
 [58] carData_3.0-5           viridis_0.6.5           DBI_1.2.3              
 [61] highr_0.11              ggforce_0.4.2           ggsignif_0.6.4         
 [64] MASS_7.3-61             DelayedArray_0.31.9     rjson_0.2.21           
 [67] HDO.db_0.99.1           permute_0.9-7           tools_4.4.1            
 [70] scatterpie_0.2.3        ape_5.8                 glue_1.7.0             
 [73] nlme_3.1-165            GOSemSim_2.31.0         shadowtext_0.1.4       
 [76] grid_4.4.1              cluster_2.1.6           reshape2_1.4.4         
 [79] fgsea_1.31.0            generics_0.1.3          gtable_0.3.5           
 [82] tidyr_1.3.1             data.table_1.15.4       car_3.1-2              
 [85] tidygraph_1.3.1         utf8_1.2.4              XVector_0.45.0         
 [88] ggrepel_0.9.5           foreach_1.5.2           pillar_1.9.0           
 [91] stringr_1.5.1           yulab.utils_0.1.4       circlize_0.4.16        
 [94] splines_4.4.1           tweenr_2.0.3            treeio_1.29.0          
 [97] lattice_0.22-6          bit_4.0.5               tidyselect_1.2.1       
[100] GO.db_3.19.1            ComplexHeatmap_2.21.0   locfit_1.5-9.10        
[103] Biostrings_2.73.1       knitr_1.48              gridExtra_2.3          
[106] bookdown_0.40           xfun_0.46               graphlayouts_1.1.1     
[109] visNetwork_2.1.2        stringi_1.8.4           UCSC.utils_1.1.0       
[112] lazyeval_0.2.2          ggfun_0.1.5             yaml_2.3.9             
[115] evaluate_0.24.0         codetools_0.2-20        ggraph_2.2.1           
[118] tibble_3.2.1            qvalue_2.37.0           BiocManager_1.30.23    
[121] ggplotify_0.1.2         cli_3.6.3               munsell_0.5.1          
[124] jquerylib_0.1.4         Rcpp_1.0.13             png_0.1-8              
[127] parallel_4.4.1          ggplot2_3.5.1           blob_1.2.4             
[130] clusterProfiler_4.13.0  DOSE_3.31.2             tidytree_0.4.6         
[133] viridisLite_0.4.2       sigora_3.1.1            scales_1.3.0           
[136] purrr_1.0.2             crayon_1.5.3            GetoptLong_1.0.5       
[139] rlang_1.1.4             cowplot_1.1.3           fastmatch_1.1-4        
[142] KEGGREST_1.45.1
```
